# The OECD and Ritualized Isomorphism: Anti-Corruption Monitoring, the ‘Narrow View’ of Corruption and the Transnational Political Order

**DOI:** 10.1177/08969205241310197

**Published:** 2025-01-23

**Authors:** Steven Bittle, Jon Frauley

**Affiliations:** University of Ottawa, Canada

**Keywords:** corruption, OECD, regulation, governance, monitoring, bribery, transnational political order

## Abstract

While there is considerable literature on the causes of corruption and its harmful effects, along with an extensive body of work focused on anti-corruptionism, there is a paucity of research that examines the anti-corruption dynamic. Few studies contemplate why anti-corruption rhetoric has come to occupy a central role in global governance, despite a growing consensus among some scholars that the anti-corruption movement has done little to reduce corruption globally. This paper explores the anti-corruption dynamic through an examination of the Organization for Economic Cooperation and Development’s (OECD’s) Anti-Bribery Convention and related peer-review process of the Government of Canada’s anti-corruption measures. It does so by situating the OECD’s work as part of a transnational movement that is more about advancing corporate interests on a global scale than about combating corruption in its various guises. In the process, our findings shed important light on how the state’s regulation of corporations is itself regulated through supranational political institutions.

Modern nation-states are constituted and constructed as ultimately similar actors under exogenous universalistic and rationalized cultural models. This produces a great deal of isomorphism and isomorphic change among them and high rates of diffusion between them and between centers of world discourse and particular nation-states (Meyer, 1999: 137).

## Introduction

The anti-corruption movement today is a massive global phenomenon. With few exceptions (e.g. Rothstein, 2011; Thompson, 2018; Warren, 2004), corruption is generally treated as bribery of a public official – typically involving an illicit transaction between two individuals, such as a government agent and an individual or individuals from the private sector – for personal gain (Barutciski and Bandali, 2015; Blyschak, 2014a, 2014b). The focus might be on supply-side and/or demand-side bribery (Barutciski and Bandali, 2015; David-Barrett, 2017), on someone using their political position to illicit bribes and thus gain a ‘private economic advantage’ (Cooley and Sharman, 2015: 11), or a company ‘gaining a competitive advantage’ (Carr and Outhwaite, 2011) by bribing a government official to secure access to resources, contracts or favourable regulations (David-Barrett, 2017). Prominent are claims that bribes undermine economic development (Loughman and Sibery, 2011) and erode trust between politicians and voters, and so companies need improved ethical cultures to combat this problem (Nichols and Dowden, 2019; also see Carr and Outhwaite, 2011).

There is now considerable literature on the causes of corruption and its harmful effects, an extensive body of work focused on anti-corruption programmes, as well as a sizable literature on white collar and corporate crime. However, as Sampson (2015) remarks, despite efforts at ‘theorizing the causes, consequences and impacts of corruption’, much less has been done in an attempt ‘to understand the dynamics of anti-corruptionism’ (p. 122). *This dynamic includes the relations and interactions through which anti-corruption programmes are formulated, diffused and institutionalized, and by which isomorphism among nation-states is achieved.*

Drawing on the literature on anti-corruptionism (Sampson, 2015) and the sociology of global capitalism/society (Carroll, 2016, 2018; Jessop, 1993; Meyer, 1999; Robinson, 2017), this paper extends beyond debates about the ‘effectiveness’ of anti-corruption measures to explore their dynamic through an examination of the OECD’s Anti-Bribery Convention and related peer-review process of the Government of Canada’s (GOC’s) anti-corruption measures. We do so by situating the OECD’s work as part of a transnational effort to diffuse a narrow notion of corruption and associated remedies that end up advancing conditions favourable to corporate interests on a global scale. Our findings shed important light on how the state’s regulation of corporations is itself regulated through supranational political institutions, where economic power works to shape political power. The diffusion of political and cultural models that place corporate power as necessarily central to national (and world) politics is central to the anti-corruption dynamic.

This paper is timely given (1) that anti-corruption rhetoric is central to global governance, but it is unclear why this is so Sampson (2015: 120); and (2) there is a growing number of critics opining that the anti-corruption movement has not reduced corruption (Andersson and Heywood, 2008; Bratsis, 2014; Engels, 2018; Heywood, 2018; Hindess, 2005, 2008; McVittie and Sambaraju, 2019; Sampson, 2010, 2015).

## The OECD and the Transnational

Given its scope and reach, the anti-corruption movement must be treated as a *transnational* movement. The dynamics of anti-corruptionism can only be fully grasped at this level (Meyer, 1999; Robinson, 2017). The most impactful of the transnational organizations, working to promote anti-corruptionism is the OECD, which represents the world’s most powerful economies. The OECD is conceptualized here as a significant source of authority and of cultural and cognitive models for its members (Meyer, 1999) and these authoritative models drive ongoing structuration of nation-states (and of local organizations and individual agents).

Founded in 1961, the OECD is an intergovernmental group that informs on social and economic policies among its member states. In the late 1980s, the OECD joined a growing chorus of voices concerned with the ‘widespread phenomenon’ of bribery, particularly the bribery of foreign public officials by corporate actors seeking to gain access to lucrative government contracts in new and bourgeoning global markets. The OECD claimed that bribery raised ‘serious moral and political concerns’ given its potential to undermine ‘good governance and economic development’ and distort ‘international competitive conditions’ (Organisation for Economic Co-operation and Development (OECD), 1997: 3)). This situation, the US business lobby argued, created an unfair playing field for businesses operating globally (Katzarova, 2019; Rose, 2015; Wedel, 2015), resulting in the OECD’s 1997 *Convention on Combating Bribery of Foreign Public Officials in International Business Transactions* (Convention). This required signatory countries to criminalize the bribery of foreign public officials to ‘obtain or retain business or other improper advantage in the conduct of international business’ (OECD, 1997: 4), with accompanying ‘proportionate and dissuasive’ penalties (p. 5). Currently, 45 countries have adopted the Convention (OECD, n.d.).

Canada realized its obligations under the Convention through the enactment of the *Corruption of Foreign Public Officials Act* (*CFPOA*) in 1999.^1^ The *Act* makes it an offence to ‘directly or indirectly’ offer a public official a ‘loan, reward, advantage or benefit of any kind’ for the purpose of influencing their duties or functions, or to ‘induce’ them to use their authority to influence the decisions of ‘the foreign state or public international organisation’ for which the official performs their duties (CFPOA, Section 3.1; Bathgate et al., 2021: 3). Individuals found guilty of bribery under the *CFPOA* are liable to fines and/or imprisonment for up to 14 years, while corporations also face possible fines as well as debarment from bidding on World Bank projects and/or Canadian government contracts for a period of 10 years (Bathgate et al., 2021: 9). As part of its ‘rigorous peer-review monitoring system’, the OECD has undertaken four reviews of the GOC’s ‘implementation and enforcement’ of the Convention. The OECD’s initial focus was the GOC’s implementation of the Convention, followed by successive assessments of efforts to raise awareness of the *CFPOA* and its enforcement. While the OECD’s (1999) Phase 1 evaluation generally concluded that the GOC had ‘met’ the requirements set out in the Convention (pp. 22, 24), subsequent assessments (Phases 2–4) have raised questions about its commitment to addressing foreign corruption and related questions regarding enforcement. As of 2022, Canada has eight convictions under the *CFPOA* (Global Affairs Canada (GAC), 2022), an enforcement record that the OECD (2023: 5) itself has referred to as ‘. . . exceedingly low 25 years after’ the law’s adoption (see Harrington, 2023).

The OECD’s official goal is to evaluate and make recommendations to signatory countries for improving their anti-corruption measures, aiming to put immense pressure by various means on member states to conform to its evolving framework. Each of the four evaluations of Canada, along with the GOC’s annual reports to Parliament outlining its anti-corruption efforts, contain information about the various rules and enforcement mechanisms relating to bribery, along with examinations of measures relating to accounting practices, anti-money laundering efforts and the promotion of social responsibility measures, among other anti-corruption initiatives.

Within the transnational political field, the anti-corruption movement is coordinated through international organizations such as the OECD. These are referred to by Robinson (2001, 2017) as ‘transnational state apparatuses’, by Jessop (1993: 23) as ‘international state apparatuses’ and by Carroll (2016: 1; 2018: 195) as ‘quasi-state institutions’. In addition to *coordinating* global capital accumulation regimes, they are important for *integrating* nation-states into this regime. This global scale necessarily transcends any one nation-state and, as it requires international coordination through ‘supranational state systems’ (Jessop, 1993: 22). This necessitates contemplation of what Carroll (2016, 2018), Carroll and Carson (2003), Carroll and Sapinski (2010, 2016), Robinson (2001, 2005, 2011, 2017) and Robinson and Harris (2000) and others call the ‘transnational capital class’ (TCC). Robinson (2017) argues that ‘the enormous concentration and centralization of capital on a global scale suggests that the global economy is acquiring the character of a planned oligopolistic structure’ (p. 177), where transnational networks are increasingly solidified due to their growing important coordinating functions. Such networks link multinational corporations and policy groups across state boundaries and vertically into local networks of power and capital (Carroll, 2016, 2018; Carroll and Carson, 2003; Carroll and Sapinski, 2016; Robinson, 2017: 177).

Carroll and Sapinski (2010, 2016) and Carroll and Carson (2003) have examined the importance of transnational policy groups for transnational capitalist class formation and although they do not directly discuss the OECD, they do discuss groups that are interlinked through it, such as the World Economic Forum and International Chamber of Commerce. These international fora provide spaces ‘where business leaders can come together to discuss issues of shared concern, to find common ground and to devise strategies for action’ (Carroll and Carson, 2003: 69). These are sites for mobilizing ‘thought leaders’ and business activists to devise ways to ‘educate publics and states on the virtues of the neoliberal paradigm’ (Carroll, 2016: 19–20; Carroll and Carson, 2003: 69). It is in these spaces – national and international – that we see ‘elite consensus building’ (Carroll and Carson, 2003: 69) or making hegemonic what might have been a ‘fractional standpoint’ (Carroll and Sapinski, 2016: 44). This is described by Katzarova (2019) in her examination of the nearly 20-year long effort by the US business community to internationalize the US Foreign Corrupt Practices Act (FCPA, 1977), culminating in the OECD’s Anti-Bribery Convention.

The OECD’s anti-corruption agenda appears contradictory in that it must work through the nation-state to regulate against corruption, while at the same time advance corporate interests on a global scale. However, when adopting a global systems view of the sort proffered by Carroll and Robinson, those contradictions disappear. Using the OECD’s evaluation of the GOC’s implementation of the Anti-Bribery Convention as our empirical focus, we discuss this in greater detail in the following two sections. The more general point is the OECD Convention and its ongoing monitoring of how well states implement it exemplifies two interconnected issues: (1) the way in which states regulate corporate behaviour is a response to developments at the transnational level of politics, which suggests (2) the state’s regulation of corporate behaviour is itself regulated through the expansion and concentration of power within supranational political institutions. As the OECD is an important supranational political organization which attempts to put downward pressure on member states to adopt its policy guidelines and anti-bribery mechanisms, our case illustrates the *importance of the transnational political field and the transnational political organization – and the global capitalist accumulation and transnational capitalist class that these represent – for shaping national and institutional governance projects, including domestic criminalization and enforcement*. While it is not our intention to suggest anti-corruption efforts are a completely fruitless endeavour, the OECD’s iterative process of monitoring and the GOC’s reports to Parliament are a window into one transnational political organization and its exerting of downward pressure on a sovereign state.

The remainder of this paper is divided into two sections. First, we further elaborate the major ideas which inform our understanding of the OECD as a transnational political organization as well as the OECD Anti-Bribery Convention and its relationship to global capitalism. We discuss the transnational political order, the supranational political organization and the transnational capitalist class to indicate how it is that states’ regulation of corporate activity is itself regulated. Second, from within this context, we discuss some *purposes* and *implications* of the Convention by attending to the OECD’s monitoring and evaluations of the GOC’s anti-bribery efforts. These include reducing corruption to bribery; protecting ‘free’ markets; perpetuating the myth of the corporation as a benevolent and responsible citizen that is constantly victimized on the world stage; and assertions that corporations can self-regulate (and can be shamed into doing so). These purposes and implications reach well beyond combatting bribery and can be seen as ideological accomplishments or effective supports for the broader view outlined.

## Supranational Political Organizations and the Transnational Capital Class

The driving force behind the OECD’s Convention – decades of US lobbying to internationalize its *Foreign Corrupt Practices Act* (FCPA) of 1977 (Katzarova, 2019; Rose, 2015) – must be understood within a broader global context wherein Keynesian Welfare states were suffering from stagflation and were being eroded or weakened as a result (Jessop, 2000, 2003). This entailed a shift in scale from national to international in terms of economic and social reproduction (Jessop, 2000: 180). As Jessop (1993, 2010: 42) notes, despite the state continuing to play a central role in economic and social life, post-welfare neoliberal states have experienced some loss of autonomy ‘through the upward, downward and sideways transfer of powers’. This includes ‘an increasingly multilateral cross-penetration of capital and the formation of a transnational capitalist class’ with states relinquishing ‘some control of its home market’ but gaining in other ways as ‘the national network becomes more transnationalized’ (Carroll, 2016: 18). In this context, states require ‘interscalar coordination’ to forge conditions that are favourable to global capital (Jessop, 2010: 42). Our immediate concern is with the upward transfer of power from states to the international political organizations that shape events extending beyond ‘formal territorial sovereignty’ (Jessop, 2010: 42). The *CFPOA* illustrates this development.

In considering the increasing interest in corruption by the international community, Bratsis (2014) notes anti-corruption is tantamount to a business strategy ‘to reduce transaction costs and to more precisely calculate its expected costs and benefits when making investment decisions’ (p. 108). This requires states to become increasingly detached from local interests ‘so as to be more hospitable to transnational capital and so as to make the policies and actions of the state much more predictable and transparent to outsiders’ (Bratsis, 2014: 108; see Carroll, 2018: 188). The detachment from local interests and shift to a global scale constitutes an upward transfer in decision-making and other capacities towards ‘supra-regional or international bodies’ such as the OECD (Jessop, 2010: 40). This is symptomatic of the ongoing restructuring of the state’s form and functions, in particular how economic and political forces are organized and directed (Jessop, 2000, 2003, 2010). At the same time, as Robinson (2001) argues, we have ‘the rise of supranational institutions’ that are both political *and* economic, not one or the other (p. 166).

States now must respond to open economies that transcend their territory in a way that is different from the days of the Keynesian welfare state (Jessop, 2000). ‘Before the end of the 1970s’, Teeple (2014) outlines, ‘national markets were appearing anachronistic, along with their political systems, in the face of the immense mass and variety that now increasingly were being produced in global economic chains’ (p. 232). In the contemporary context, states are not as powerful in regulating and reproducing economic relations. They are subjected to supranational political entities that steer them mainly through ‘soft’ law guidance (Rose, 2015). Shifts such as these, according to Jessop (1993), ‘correspond to long-term structural changes in the global economy’ and are ‘associated with the blurring of the state’s boundaries and its growing involvement in decentralized societal guidance strategies rather than centralized imperative coordination’ (p. 10). This greatly expanded role of supranational organizations in economic and social reproduction has ‘undermined’, ‘hollowed out’ or ‘weakened’ the territorial sovereignty of states with respect to the goals of global capital accumulation and social reproduction (Jessop, 2000: 180, 2010: 43). Here the state struggles to retain autonomy within a globalized economy that aims to homogenize domestic economic policies and remove trade barriers (Robinson, 2001: 160). ‘National economies’, argues Robinson (2011):

have been reorganized and reinserted as component elements of his new global production and financial system . . . [different] from that of previous epochs, when each country had a distinct national economy linked externally to one another through trade and financial flows. This is a shift from international market integration to global productive integration. (p. 353)

In this post-welfare circumstance where ‘corporations have the structural power to play one national workforce off against another’ and where ‘giant corporations and massive pools of money capital concentrate enormous social power in the capitalist class’s top tier’ (Carroll, 2016: 13), states must participate in a globalized economic and political regime and become less concerned with closed domestic or local economies. Supranational governance systems become more prominent and take on new functions (Jessop, 1993: 22). What is significant is the power of the already corporatized nation-states of the world’s most powerful economies was eroded as the conditions for global market-capitalism were expanded. Much of this weakening of nation-states as they transitioned from Keynesian welfarism to what today is commonly referred to as neoliberalism was taking place at the same time as the neoliberalized ‘development’ of the ‘third world’ through the lending policies of the International Monetary Fund (IMF) and the World Bank (WB). These groups forced neoliberal structural adjustment of impoverished states in order to integrate these economies into a global production and financial system. Policy groups such as the OECD ‘seek to persuade’ and ‘operate at one remove from the structural adjustment programs’ (Carroll and Carson, 2003: 99).

These groups constitute, according to Meyer (1999, 2010), ‘significant others’ and ‘consultants’ that ‘instruct and advise actors on how to be better actors in light of general principles’ (Meyer, 2010: 7). The actors instructed (i.e. nation-states) are entities constructed through globally diffused cultural meaning systems (such as neoliberalism) as sovereign ‘empowered actors’ (Meyer, 1999, 2010; Meyer et al., 1997). Diffused cultural and cognitive models operate as a ‘cosmology or as a cultural canopy establishing principles that the world can be understood in an integrated and standardized way by everyone in common’ (Meyer, 2010: 8).

These policy organizations form a superordinate and constitutive cultural control network immensely important for providing ‘a set of cognitive models defining the nature, purpose, resources, technologies, controls and sovereignty of the proper nation-state’, which then operates as an ‘exogenous control’ in regulating ‘nation-states and their activities’ (Meyer, 1999: 123). They play an awareness-raising and shaming role if member states do not conform (discussed below). The rapid diffusion (Meyer et al., 1997; Strang and Meyer, 1993) of these cognitive models between similar nation-states (such as OECD members) and to peripheral developing states has served to open new markets for ‘foreign investment’ (i.e. wealth extraction by multinational corporations). Importantly, these significant others that advise nation-states and state agencies ‘parallel high priests in a knowledge-society’ (Meyer, 2010: 10). This dovetails with Wedel’s (2015) argument that anti-corruptionism is viewed akin to ‘a gospel of enlightened capitalism’ with central bankers and economists taken to be its high priests (p. 4). Anti-corruptionism, according to Sampson (2010), is ‘a globalised elite discourse’ that has fueled a veritable industry.

According to Robinson (2011), imposing structural adjustment on developing economies is one of the key developments facilitating such diffusion (p. 353). This enabled incorporation of regions that might have remained outside or only weakly integrated into a global accumulation and financial regime. At the same time, it was necessary to create a ‘global legal and regulatory structure to facilitate what were emerging globalized circuits of accumulation worldwide’ (Robinson, 2011: 353). The transformation of capitalist states was situating these latter as crucial nodes and supports of an expanding corporate power. The OECD’s anti-bribery Convention is but one example of this.

Fulfilling their obligation to the OECD to restructure their domestic legal systems, member states crafted their own anti-bribery laws and in so doing worked to facilitate nationally the supports necessary for an emerging transnational capitalist class. Robinson (2011) asserts that states are inserted into a global order of accumulation, and it is this that is key to understand (Carroll and Sapinski, 2016: 40). *This transnational context and implications for sovereign state decision-making is something that criminologists have failed to adequately grasp because they are overwhelmingly wedded to the idea that criminalization and crime control are national in scope, ordered and organized by a national state.* According to Robinson (2001: 159, 2005, 2017: 181), contemporary economic and political developments cannot be understood ‘in the context of such state-centric and nation-centric paradigms’. This is because transnational governance systems consist of ‘a loose network comprised of supernational political and economic institutions *together with* national state apparatuses that have been penetrated and transformed by transnational forces’ (Robinson, 2005: 317). This applies equally to thinking about how and why states regulate corporations in the way they do: this is done by states as integrated into a global system and pressurized accordingly.

There is no doubt that states, in creating their own criminal and regulatory law, are responding to local circumstances, but criminologists overwhelmingly fetishize the local, regional and sometimes the national, to the degree that they fail to understand the existence and impact of a transnational political order and downward pressure exerted over national and regional developments. Some criminologists have attempted to make inroads in this direction by examining the state’s ‘symbiotic’ relationship with corporate power, acknowledging an international political sphere (Kauzlarich and Kramer, 1993; Kramer et al., 2002; Michalowski and Kramer, 1987; Pearce and Tombs, 1998; Tombs, 2012). However, these scholars have not fully developed how this state–corporation nexus is today more than at any other time in history facilitated and reproduced through downward pressure exerted by a transnational capitalist class working through supranational political organizations to maintain, extend and enhance globalized neoliberal market relations. We argue that what needs to be conceptualized further is how this transnational space represents a *political order characterized by supranational governance networks* to which some of the powers of former welfare states have been transferred and from which these supranational political entities and a transnational capitalist class operate to coordinate and coerce developed and developing states to align their economies to make wealth extraction less costly for corporate entities. Criminalizing the bribing of government officials is one way to reduce costs. Nation-states pick up the tab for making corporate capitalism more lucrative.

Although it appears that the OECD monitoring process is to help states implement better the Convention to regulate bribery internationally, and it appears a unified stance against bribery is an inherent good, it exemplifies how supranational political organizations exert downward pressure on states to regulate corporate behaviour (i.e. how states’ regulation of corporate behaviour is itself regulated) in the business-friendly ways they do. This downward pressure can be a very positive thing, for example, in the case of policing human trafficking or a desire to strengthen democracy internationally. However, it is imperative to bear in mind that when speaking of the OECD and anti-corruption, we are speaking of political and economic transformation that mainly concerns corporate actors, policy coordination, publicity and business activism towards the expansion of corporate wealth extraction and reducing private-sector costs associated with this. Far from holding corporations to account for their misdeeds, the Anti-Bribery Convention and Canada’s *CFPOA* facilitate removing barriers and costs for corporate pursuit of profit that will, in turn, inevitably involve *more* not fewer harmful acts.

We have thus far discussed how states’ regulation of corporate behaviour itself is regulated. The OECD Convention and monitoring efforts help to promote the conditions for this. We can see the effective supports for this in the several purposes and implications of the Convention and monitoring efforts that reach well beyond the goal of combatting bribery. The following section examines some examples of this within the context of the OECD’s evaluation of the GOC’s anti-bribery efforts.

## Purposes and Implications Beyond Bribery: Isomorphism and Effecting Supports for Global Capitalism

The OECD’s monitoring of the GOC’s anti-corruption law and policy serves several mutually reinforcing *purposes*. Importantly, as critical socio-legal scholars remind us, when examining law, it is essential to look beyond its coercive function, if not only because it barely exists at the top end of the social hierarchy, but because law is not simply a body of external rules that acts directly on its target. Law has an important productive or ideological role in (re)producing social relations (Brabazon, 2016a, 2016b; Hunt, 1993). Hunt (1993) argues, a la Gramsci, that law is a form of ideological domination in that it conveys a ‘complex set of attitudes, values, and theories about aspects of society’ and can serve to secure consent to these. Law gives *effect* to social relations in attempting to smooth over the very contradictory and disordered relations and struggles that are in the backdrop (Hunt, 1993: 121). There is, in other words, effectivity to legal ideology – consensus around something such as ‘corruption is bribery’ or ‘corruption is bad’ is ‘produced and mobilized’ through ideological domination (Hunt, 1993: 53). It is not predefined but achieved. ‘The normative content of law provides authoritative definitions of social relations’ (Hunt, 1993: 54). This is important given the transnational origins of anti-corruption law. In addition, law has always been an important mechanism for forcibly subordinating actors to market relations such as waged labour for subsistence and subjecting some to violent coercion if they should attempt to circumvent the sanctioned means installed to facilitate this and simultaneously reproduction of capitalist social and economic relations (Gordon, 2009; LeBaron and Roberts, 2010; Roberts, 2017; Robinson, 2020).

Law is a vehicle for diffusing values, norms and practices from the transnational to the national arena. It provides one avenue of diffusing globalized cultural and cognitive models of structural organization and social action that play into isomorphism between states (Meyer et al., 1997: 149). As Hunt (1993) remarks, ‘the real power and influence of law in capitalist society’ (p. 32) is found in its dual functioning: its practical effectivity of concretizing dominant interests as everyone’s interests, and its ability to provide ‘justification or legitimation for these interests in terms of some higher and apparently universal interest of all’. From this vantage point, anti-corruption law is important to the (re)production and transformation of social relations (Brabazon, 2016a, 2016b; Hunt, 1993). The universal interest *appears* to be corruption, but it is what this represents in the global arena on a more fundamental level: the costs of wealth extraction, corporate values and norms.

### Narrowing Corruption to Bribery

The OECD Convention on bribery and related monitoring efforts (re)produce a narrow definition of corruption, which reduces the corruption problem to a largely technical matter requiring the right mix of tools and enforcement. It reinforces the idea that corruption is a narrow set of behaviours concerning bribe taking by greedy government officials in other countries who are willing to break the law. Current debates about the ‘new normal’ of corruption tend to draw from narratives about the changed character of individuals, who have become greedier, or whose moral compass has gone awry (Wiegratz, 2015: 50). Corruption from this perspective ‘. . .is generally seen as a kind of pathology’ (Miller, 2015: 61).

This idea that corporations are victims, not offenders, and that corruption is the result of individual greed *obscures the fact that the OECD anti-bribery convention leaves corporate power relatively unchecked* and works towards the protection of ‘free’ markets on behalf of corporate wealth extraction. Bribery is simply seen as an opaque cost of doing business that must be eliminated as it dissuades foreign investment. This individualizes the corruption ‘problem’ and downplays the role of law itself in creating the conditions for corporations to dominate markets and public interest globally (Galanis, 2020).

An individualized account also emerges from the assumption that corruption is a ‘public sector problem’ in which there is too much decision-making authority in the hands of government officials, leading to corrupt practices. The WB definition of corruption focuses on the ‘abuse of public office for private gain’, creating a false distinction between public and private and leaving the impression that corruption is only problematic when it enters the public realm. It is the idea that the state has been captured by private interests, and weak governance is therefore the culprit (Whyte, 2015: 6–7). There is, thus, an overall tendency to put the onus on so-called developing countries – corruption is not about us, but them (the ‘others’).

Corresponding to the idea of corruption, as a problem of greedy individuals, not of corporations or systems and institutions, OECD evaluators raised concerns about the role of organized crime in cases of corruption. As a remedy, they recommend that officials at the Canada/US border be made aware of possible bribes from criminals involved in smuggling operations (OECD, 2004: 8), raising the spectre that conventional criminals are infiltrating an otherwise efficient system. The individualizing effect also emerges in the discussion of whether the *CFPOA*’s reference to ‘for profit’ was clear enough to include non-profit organizations. As the OECD’s (2004) Phase 2 report notes, the CFPOA’s bribery offence ‘. . . applies in respect of a person who bribes “in order to obtain or retain an advantage in the course of business”’, and section 2 defines ‘business’ as ‘any business, profession, trade, calling, manufacture or undertaking of any kind carried on in Canada or elsewhere for profit’ (p. 28). As the lead examiners note, ‘. . . the term “for profit” in the CFPOA is very unclear’ and express concern ‘. . . about the possibility of the non-application of the CFPOA to non-profit companies’, resulting in ‘the non-coverage of a sizable sector in the Canadian economy’ (OECD, 2004: 30). While the report also notes that some for-profit companies might hide their bribes within non-profit transactions, the dominant focus obscures the link between the profit motive of corporations and corruption.

A narrow, legalistic approach also downplays the role of corporations in both causing corruption and benefitting from anti-corruption(ism). Nyberg (2021) is critical of definitions that fixate on ‘quid pro quos’ and instead focuses on the corruption of democracy by corporations and corporate interests. Corruption is not episodic, from his perspective, but rather normalized through the efforts of powerful corporate actors to infiltrate the political realm. This creates a profoundly anti-democratic scenario that is ripe with corruption, or what Nyberg (2021) likens to government policymaking being ‘“captured” by private business interests’ (p. 3). Whyte (2015) also draws attention to the ‘distortion of the public realm by private interests’ (p. 6). For Whyte (2015), ‘[c]orruption can be understood as part of ‘the neo-liberal harvest’, in which unrestrained self-interest and aggressive economic self-maximisation are constructed as the logical aims of economic policies’ (p. 10). Focusing on corruption in ‘weak’ states therefore obscures the class interests at play in the institutional corruption throughout the Global North.

Critical scholars also point to the very structure of corporations and the state’s support of this legal fiction as being at the heart of the corruption problem (Glasbeek, 2002; Tombs and Whyte, 2015). For Galanis (2020), as capitalism took root – and with it the proliferation of ideas around the ‘free market’ and the inherent good of economic ‘progress and growth’ – the entire idea of a ‘market society’ took precedence to the point that it is now something that almost completely subjugates ‘private goals to private economic ones’ (p. 298). Corruption is therefore not the result of flaws or a breakdown in otherwise efficient and effective political and economic systems, but instead a product of routine practices that serve to maintain and extend ‘the power of corporations, governments and public institutions’ (Whyte, 2015: 5).

### Protecting ‘Free’ Markets (Neoliberal Capitalism and Wealth Extraction)

A significant theme emerges in relation to the *CFPOA’s* overall purpose to protect free markets. As every GOC annual report to the Parliament baldly states, the Convention ‘aims to stop the flow of bribes and to remove bribery as a non-tariff barrier to trade, producing a level playing field in international business’. In this sense, it is important to recall that a driving force behind the Convention was US-led claims of unfair competition for American companies that were subjected to their *Foreign Corrupt Practice Act* (*FCPA*), while companies from other jurisdictions without such laws were free(er) to run amok (Katzarova, 2019). The Convention is thus rooted in concerns with *corporations being victimized* by other companies that do not play by the rules.

For instance, following a discussion in the Phase 2 Report of the foreign trade by Canadian companies, including US$3 billion of commodity exports to China in 2001 and another US$940 in ‘Canadian direct investment’ in Russia in 2000, the OECD (2005) notes ‘. . . a potential for Canadian companies engaging in foreign trade to be exposed to demands for bribes’ (p. 9). The OECD (2011) further states this observation was ‘. . . confirmed by a lawyer for small and medium enterprises (SMEs) who stated that in his experience SMEs involved in foreign trade are encountering these kinds of situations’ (pp. 5–6). Likewise, there is reference to Canada’s export credit agency, Export Development Canada (EDC), and its efforts ‘to inform customers of the potential risks they face if exposed to corrupt business practices, and to encourage the development of corporate best practices in this area’ (OECD, 2003: 3). The same goes for the extractive industry, which despite its sordid reputation, is positioned as a victim of corruption. As the OECD’s Phase 3 (OECD, 2011) report states:

[h]aving regard to the strength of the Canadian extractive industry, it is notable that representatives from that sector, and from civil society, explained during the on-site visit that high risks of bribe solicitation are present in a number of countries where the extractive industry operates. (p. 8)

In the same report, a representative from ‘one of Canada’s most well-known and respected companies globally’ states that despite Canada’s reputation as an honest society, we should not forget that Canadian companies operating abroad ‘. . . face the same pressures as companies from other countries to engage in corrupt practices’ (OECD, 2011: 9). At worst, these claims characterize corporations as victims of bribery; at best they position them as passive players in the corruption phenomenon, only becoming involved when subjected to the demands of corrupt foreign governments and individuals.

Further indications that facilitating trade and advancing economic interests are the Convention’s underpinning motivation emerges in the GOC’s reporting of its anti-corruption measures. Take, for instance, the introduction of the *Extractive Sector Transparency Measures Act* (*ESTMA*) in 2015, which the GOC claims as a key measure in ‘global efforts to deter corruption and promote transparency in the extractive sector’ (GAC, 2015: 6). The *ESTMA* requires extractive companies to ‘report payments including taxes, royalties, fees, and production entitlements of $100,000 or more to all levels of government in Canada and abroad’. However, the Canadian extractive industry has long been plagued by allegations of corruption, environmental degradation and various human rights abuses (Gordon and Weber, 2008, 2016). Despite these allegations, the GOC’s goal is to ensure Canada’s extractive industry continues to ‘prosper and to provide the *broad economic benefits* that are fundamental to Canada’s success’ (GAC, 2015: 6; emphasis added).

The debate around facilitation payments also speaks of the Convention’s underlying concerns with the smooth flow of capital. Originally, the *CFPOA* exempted facilitation payments, such as a ‘loan, reward, advantage or benefit . . . made to expedite of secure the performance by a foreign public official of any act of a routine nature that is part of the foreign public official’s duties or functions’ (OECD, 2011: 14). Although repealed in 2017 after the OECD (2011) expressed concerns the Canadian Revenue Agency was ill-equipped to differentiate between a ‘reasonable expense’, ‘facilitation payment’ and a bribe (p. 48); the fact that this exemption was originally part of the *CFPOA*, as well as the nature of the debate which ensued, reveals the underlying economic motivations. On one level, its original inclusion was expressly about small payments that helped ‘speed up’ a process in which it was the ‘official’s job to do’ (OECD, 2004: 27). Likewise, during the OECD’s evaluation process, some representatives from the business sector suggested it was common for ‘companies to make a payment to expedite or secure the performance of some act by a foreign public official, and that facilitation payments are rarely recorded in corporate books and records’. Accountants and auditors who participated in the OECD’s (2011) review process agreed and stated they paid little attention to facilitation payments when auditing a company because ‘those payments usually do not materially affect the corporation’s financial statements’ (p. 14). In many ways this discussion is about what constitutes a tolerable level of bribery – that is, when greasing the wheels goes too far and perverts the ‘free’ market beyond reason.

Viewing corruption narrowly as bribery and holding corporations to be victims of corrupt state officials speaks of how states have downplayed corporate harms. In effect, the anti-corruption agenda helps maintain the ‘favourable political terrain’ (Jessop, 2010: 42) for corporations to exploit profit-making opportunities on a global scale – the very pathways that create the conditions for orthodox corruption to occur via so-called free markets that are highly competitive and lucrative and in which companies can and will do what it takes to get ahead. In this respect, paradoxically, anti-corruption(ism) helps (re)cement the very conditions that underpin the corruption phenomenon.

### Myth of the Corporation as Benevolent and Responsible Citizen: Corporate Victimization and Corporate Social Responsibility

The various anti-corruption measures that flow from the *CFPOA* and OECD evaluations (re)produce the belief that corporations are inherently good and rational entities capable of functioning within the confines of the law and without unnecessary government intervention. As such, while punishing the odd corporate miscreant (i.e. bad apples) is expected, the predominant focus is investment in corporate social responsibility (CSR) and spreading awareness of the *CFPOA* to ensure corporations and corporate actors ‘do the right thing’. It thus reproduces the myth of the corporation as a friendly, responsible citizen capable of self-regulation (Tombs and Whyte, 2015).

CSR is a significant point of discussion throughout the OECD’s monitoring process. For instance, in their Phase 2 report, the OECD praises Canada for promoting

corporate social responsibility, which includes counselling Canadian businesses against engaging in foreign bribery, to the range of services provided by the Trade Commissioner, and the DFAIT intranet website (Horizons) now provides information to Canadian trade officers on how to counsel businesses abroad on the CFPOA and the risks of bribery (OECD, 2004: 7).

The idea that companies are inherently responsible and can self-manage CSR expectations is further evidenced by positive reviews in the same report of the large Canadian companies with codes of conduct that refer to the CFPOA (OECD, 2004: 10).

CSR is also a significant component of the OECD’s (2011) Phase 3 evaluation. As the lead examiners recommend:

*. . . the implementation and enforcement of internal controls, ethics and compliance programmes by Canadian companies . . . is integral to the global fight against foreign bribery. While current efforts to promote corporate social responsibility go some way to encouraging the development of internal company controls, the merging together of corporate social responsibility programmes, which are encouraged but not legally required, and compliance programmes designed to prevent and detect CFPOA violations, has caused some confusion among the private sector. The lead examiners therefore recommend that Canada take steps to increase focus on promoting compliance programmes and measures specific to the CFPOA . . .* (pp. 47–48; emphasis original)

The theme continues in the OECD’s most recent evaluation, with Transparency International Canada’s (TI Canada, 2023) submission to the lead examiners promoting ‘alternative avenues to make corporations and individuals accountable for wrongdoing and to reduce incentives to engage in corruption’ (p. 26). As part of this, TI outlines the potential of beneficial ownership registries, the *ETSMA*, Canada’s integrity regime that includes debarment for those found guilty of corruption (although they raise concerns about the 10-year debarment being too rigid and it should be more flexible to allow for context/specific facts of a case), and stakeholder engagement. When it comes to stakeholder engagement, TI recommends civil society work with governments and the private sector to address ‘corruption risks’. Cited is the example of the Maritime Anti-Corruption Network, which is an industry-led effort to address corruption in the shipping industry, as what can be done – ‘to use a collective voice for improved integrity in ports around the world’ (TI Canada, 2023: 31).

Discussion of CSR is most prominent starting in 2006, when the GOC reports on Foreign Affairs and International Trade (DFAIT) Canada’s CSR Roundtables. As the GOC states, this initiative was introduced so ‘government, NGOs, labour organizations, businesses and industry associations’ could examine ‘. . . ways to strengthen approaches to managing the external impacts of international business activities to benefit both businesses and the communities within which they work’. It emphasizes that discussions at the Roundtables will ‘touch on a wide range of CSR-related issues including corruption’ (2006: 9). As a result, the GOC announced a new CSR policy in 2009 that (once again) focuses on the extractive industry, *Building the Canadian Advantage: a CSR Strategy for the Canadian International Extractive Sector*. From the GOC’s perspective, the policy’s purpose is to ‘help’ Canadian extractive companies manage their ‘social and environmental risks’ when operating abroad. The policy contains four pillars, including supporting ‘host country capacity-building’, promoting ‘international CSR’, introducing an ‘Extractive Sector CSR Counsellor’ and developing a ‘CSR Centre of Excellence’.

CSR is thus positioned as a key cog in the anti-corruption wheel despite little empirical evidence of its efficacy (Faucet, 2006; Fleming and Jones, 2013). Furthermore, when it comes to the OECD’s monitoring process, CSR serves an ideological role in promoting corporations as rational organizations capable of self-regulating, while at the same time downplaying the role of the state as a central figure in regulating corruption. In fact, the GOC’s promotion of CSR, coupled with what the OECD (2023) characterizes as an ‘exceedingly low’ *CFPOA* enforcement record (p. 5), demonstrates how the monitoring process *actively removes the state* from responsibility for the corruption ‘problem’ in favour of measures that promote the self-regulating interests of Canadian businesses abroad.

### Awareness and Shaming

Rooted in beliefs that corporations are rational and motivated to operate within the confines of the law, the OECD’s monitoring process also emphasizes ‘raising awareness’ as key to implementing the Convention and preventing corruption. The Phase 2 report, for instance, stresses the need for the GOC to improve awareness of the *CFPOA* across all government departments responsible for oversight of the Convention (e.g. the Justice and Foreign Affairs), as well as in the areas of enforcement and agencies most likely to come into ‘contact with companies engaging in business abroad’. It also calls on the GOC to monitor this awareness (OECD, 2004: 10). The federal government’s response to the Phase 2 evaluators was they were working hard to spread awareness of the law throughout the responsible departments, who subsequently spread the information to each of their relevant stakeholders, including private companies. In turn, there are claims that private companies, particularly small and medium-sized enterprises (SMEs) simply do not know about the law, and if they did, they would perhaps avoid situations in which they are asked for a bribe. EDC is cited approvingly as posting ‘. . . information on its website about corruption and bribery, including the CFPOA, the Convention, and the OECD (2004) Export Credits Group Action Statement on Bribery and Officially Supported Export Credit’ (p. 9). Meanwhile, a lawyer of SMEs states that most of his clients are unaware of the law, and those who are ‘aware of the CFPOA perceive that there is a small risk of being punished’ (OECD, 2004). Once again, the message is that if companies know the law, they will not get involved in bribery.

All the OECD reports and the GOC’s annual reports to Parliament are technocratic in orientation by routinely including certain indices that conform to the OECD monitoring processes rather than offering serious consideration of corruption as a national or global problem. Each report includes sections on enforcement, prosecutions, awareness-raising and monitoring information, along with updates about the ratification of the Convention in other jurisdictions. This reporting mechanism, along with the OECD’s monitoring, creates a feedback loop in which the OECD’s vision of corruption and anti-corruption(ism) is reinforced. An example of this is the GOC’s response to the OECDs recommendation to raise further awareness of the *CFPOA*, in which the GOC lists more than a dozen different awareness-raising activities as indications of their anti-corruption efforts, including presentations at conferences and meetings, specialized training, updated polices and newsletters. On one hand, raising awareness is seen as necessary for ensuring people know the law so that they can avoid running afoul of it. On the other hand, it is based on certain assumptions that there are individual bad apples operating in an otherwise good and efficient system, and we therefore need to raise awareness about these situations so that the ‘good’ people can root out the ‘bad’. What is more, reporting these awareness-raising activities is relatively risk and consequence free, as there is no way to determine their effectiveness or to link them to enforcement activities, especially in the face of such few charges and convictions. It gives the illusion that something is being done to combat corruption without having to prove otherwise.

Raising awareness is linked to shaming. Awareness puts the failings observed in the monitoring process into the public spotlight. This puts pressure on states to ‘do something’. Countries are thus shamed into implementing and enforcing the Convention and to regulate corporate behaviour in lockstep with other member states, which are also in lockstep with the needs of the multinational corporations to reduce the costs of wealth extraction, particularly as concerns developing markets. Although cooperation could possibly be elicited from the WB, IMF and other financial institutions, the OECD itself has no coercive mechanisms to force states to comply with the Convention. Instead, it employs something akin to Braithwaite’s ‘reintegrative shaming’ (Ayres and Braithwaite, 1992; Braithwaite, 1982, 1989; Braithwaite and Fisse, 1985, 1987). It is an approach that emphasizes persuasion and education (i.e. awareness) to ensure organizations comply with regulations. This is to adopt the belief that individuals are ‘reasonable, of good faith, and motivated to heed advice’ (Braithwaite, 1989: 131), and that corporations and corporate actors are not ‘true’ criminals (Pearce and Tombs, 1990, 1997, 1998; Tombs and Whyte, 2007) Rooted in rational actor theory, Braithwaite’s pyramid of regulation advocates persuasion first, providing corporations with opportunities to learn from their mistakes and take the necessary corrective action. This approach avoids the assumption that all corporations are potential offenders; it reduces the defensiveness and resistance of corporations by turning to education and persuasion instead of punishment (Braithwaite, 1989: 132–133). Without abandoning punishment strategies, he cautions against an over-reliance on criminal laws since they are rarely enforced and fail to deliver deterrence (Braithwaite, 1989: 150; Pearce and Tombs, 1997: 96–97).

For those corporate crime scholars who view private, for-profit corporations as inherently criminogenic (Pearce and Tombs, 1998; Tombs and Whyte, 2015), compliance models of regulation ignore the reality that corporations simply will not self-regulate in the absence of external pressures (Slapper and Tombs, 1999: 184). Given that the OECD’s monitoring process does not make sanctions available, one wonders if awareness and shaming are enough to motivate compliance. In this sense, while cooperative models tout flexibility as consistent with market efficiency, in the end they work to the advantage of powerful corporate interests (Noble, 1995: 271–272), effectively ignoring how the organizational imperative to accumulate maximal profits can and does take precedence.

These specific *purposes* and *implications* all work towards producing isomorphism among states. The OECD Convention offers a model of good governance – cultural and cognitive models – made available to states for use in conforming to ‘best practices’ not only for what is defined as ‘good’ governance but also what it means to be a successful capitalist democracy rather than a failed one. It goes without saying that state compliance with collectively agreed upon standards requires a host of ‘functionaries and offices that are direct reflections of world institutions and professions’ (Meyer, 2010: 12). As standards for these professions change at a global level, so too will practices change at the local level. Thus the ‘industry’ in anti-corruption as much as the OECD Convention is key for creating isomorphic political and economic conditions among states that are favourable to corporations.

## Conclusion

This paper has demonstrated many things, but one major issue is that laws as they apply to powerful corporations are rarely about controlling or punishing corporate offenders. Moreover, these laws and related policies and practices serve a more insidious and ideological role. They are much more than simply illusions of control; they are productive in helping carve out, maintain and reinforce the conditions within which companies exploit globally. In the end, to paraphrase Pearce and Tombs (1990), the OECD’s anti-corruption efforts are based on the inherent belief in the legitimacy of corporate capitalism and the illegitimacy of policing it.

Many of the laws and policies implemented at a national level, such as anti-bribery laws, originate in the transnational domain first as either binding legal norms or simply as strongly advised guidelines (Rose, 2015; Shelton, 2009). States then may decide or be pressured to incorporate these into their domestic legal regimes. This is the case with the OECD Convention and recommendations resulting from the monitoring process. Although recommendations do not have the same legal status, they both have nevertheless greatly impacted Canada and other member states. As Rose (2015) concludes, the OECD Convention ‘reflects the relatively narrow interests’ of a ‘relatively exclusive group of states’ (p. 218). These states are major capital exporting economies and are home to the world’s most powerful and influential corporations. The monitoring process of the OECD’s Convention has had a profound impact on pressuring member and non-member states to accept the narrow definition of corruption as bribery and to enforce this, giving effect to a fictive reality, while ignoring undue influence by corporate power on democratic (and other forms of) decision-making and the expansion of global capitalism and a transnational capitalist class. Bureaucratic corruption has been criminalized but the Convention is silent on political corruption. In addition, OECD monitoring is not limited to examining how well states have implemented the Convention. It also monitors compliance with non-binding recommendations that accompany the Convention, and which change over time. It is this ‘soft law’ that is the ‘standard form for norm-creation at the OECD’ and its legitimacy is questionable, particularly as these norms enter into and restructure the social, economic and political infrastructure of member states (Rose, 2015: 218).

States are important and active partners for realizing social, political and economic policies required to support the Convention. State-law that implements the Convention at a national level takes an *economic* policy position in that these laws criminalizing bribery are oriented to the needs of the private sector (corporate commercial activity). The express concern is with ensuring a uniform playing field for all corporations participating in wealth extraction (i.e. foreign investment) in economies outside the powerful capitalist economies of the Global North. These target economies include the former Soviet states as well as those of so-called underdeveloped and poorer states. We can also view the Convention as *social* policy as it is expressly concerned with the (re)distribution of wealth/capital. Agents of ‘foreign’ governments are not entitled to a share in this capital or in profits from Western investment and any corporation that distributes capital in this regard will be punished. Thus, the law sets out what is a legitimate and illegitimate form of distribution of resources. The Convention is concerned also with *political* policy as it requires member states to criminalize bribery of foreign officials by agents of corporations. To enact criminal law (i.e. law-making and criminalization), and to allocate dedicated state resources to engage criminal enforcement and prosecution as well as adjudication by criminal courts is a political policy decision.

There could be a great many reasons for why OECD member and non-member states have implemented and (not) enforced its anti-bribery norms. This is an empirical question that has not been well-answered (Rose, 2015: 219–220). But as Carroll (2018) and Robinson (2011), and others have shown, there has been growing business activism since the 1980s to promote and consolidate global neoliberal capitalism and to link and integrate economies and economic elites worldwide. The global anti-corruption movement factors into this growth, expansion and consolidation. Since many of the economies of concern are ‘foreign’ and beyond the territories of the most powerful and influential capitalist democracies whose corporations are those of primary concern to world commerce, and as the OECD represents these powerful states and their giant firms, supply-side criminalization is seen as the only viable solution for removing bribery as an opaque transaction cost and to reduce the ‘victimization’ of corporations by corrupt public officials. The singular focus on bribery concerns what is seen as a barrier to entry to foreign markets in an era where economies globally have been liberalized and many industries privatized and deregulated.

Our analysis does not contend that anti-corruption efforts are only a fool’s errand. However, when the OECD Convention and monitoring practices are scrutinized, it becomes apparent that ‘corruption’ is not the object of regulation. In effect, anti-corruption laws and related enforcement and policy efforts are manufactured in a manner that artificially and strategically narrows the problem of corruption to bribery. There is a significant ideological dimension that reinforces a particular vision of states as autonomous, corporations as benevolent victims of greedy state officials, and of free markets as the key to social and economic development and for the spreading of democracy. These are all, paradoxically, at the heart of a corruption problem that the Convention and monitoring are said to address.

If corporate crime scholars are correct about the criminogenic structure of the corporation, that these organizations do produce and engage in a wide variety of socially injurious actions (Michalowski and Kramer, 1987; Pearce, 1976; Pearce and Tombs, 1998; Tombs and Whyte, 2015), and if as we have argued the Convention facilitates removal of opaque transaction costs to enable more lucrative corporate plundering while securing the conditions for a global capitalist accumulation regime, then the Convention’s narrow view of corruption likely will facilitate a wide variety of socially injurious actions.

## Notes

1. Canada utilizes an articulated approach to combat bribery, leveraging its massive professional civil service to spread responsibilities (and accountability) over 12 agencies and departments, but without any direct coordination. Some of these agencies enforce Federal legislation that aligns with and/or supports Canada’s *CFPOA.* Given space constraints, we can only name these agencies/departments rather than explain their role in the enforcement of Canada’s anti-bribery regime. The basic point is that Canada’s approach to corruption is highly bureaucratized, fragmented and, as the OECD suggests, ineffective. Canada’s articulated approach is thus: (1) for direct enforcement of the *CFPOA*, the Royal Canadian Mounted Police (RCMP) is utilized; (2) prosecution under the *CFPOA* is handled at the Federal level by the Public Prosecution Service of Canada (PPSC); (3) intelligence is gathered by the RCMP and FINTRAC (Financial Transactions and Reports Analysis Centre of Canada); (4) the international trade and management unit, Global Affairs Canada, liaisons with the OECD and other international bodies and enters into international partnerships concerning anti-corruption (and other matters) related to trade and business relations; (5) Export Development Canada (EDC) is essentially a publicly funded money lending agency for promoting and facilitating Canadian businesses expanding abroad; (6) Canada Revenue Agency (CRA) aids in deterring bribery of foreign officials using tax law and international tax agreements with other states’ agencies; (7) the Department of Justice handles deferred prosecution agreements (DPAs) wherein companies guilty of wrongdoing can be diverted from criminal prosecution so as not to face debarment from World Bank or Canadian Government contracts (https://www.tpsgc-pwgsc.gc.ca/ci-if/ar-cw/documents/aps-dpa-eng.pdf); (8) Canadian Commercial Corporation (CCC) is Canada’s ‘government to government contracting agency’ supporting Canadian commercial enterprises’ relations with other countries; (9) Public Services and Procurement Canada (PSPC) ‘supports federal departments and agencies in their daily operations as their central purchasing agent, real property manager, treasurer, accountant, pay and pension administrator, integrity advisor, common service provider and linguistic authority’ (https://www.canada.ca/en/public-services-procurement.html); (10) Competition Bureau of Canada (CB) enforces four Federal pieces of legislation to stem anti-competitive practices and to encourage competition; (11) Treasury Board Secretariat (TBS) is oversight regarding spending of government departments and agencies and (12) Natural Resources Canada (NRCan) is responsible for regulating and ensuring sustainable use of Canada’s natural resources and functions as oversight of the extractive sector within Canada.
